# Reflective groundcovers promote anthocyanin content and advance fruit maturity of ‘Evercrisp’ apples grown in the Mid-Atlantic US

**DOI:** 10.3389/fpls.2024.1478498

**Published:** 2024-10-24

**Authors:** Md Shipon Miah, Macarena Farcuh

**Affiliations:** Department of Plant Science and Landscape Architecture, University of Maryland, College, Park, MD, United States

**Keywords:** reflective groundcovers, skin blush, Malus domestica Borkh, anthocyanins, gene expression

## Abstract

Enhanced skin blush is critical in many apple cultivars to ensure crop profitability and acceptability. Anthocyanin content is a crucial determinant of apple skin blush. Reflective groundcovers are a preharvest management strategy that can improve apple skin blush, but integrative studies assessing its effects at the environmental, physiological, gene, and metabolite levels are lacking. In the present study, we assessed the impact of reflective groundcovers on light environment, preharvest fruit drop, internal ethylene concentration (IEC), fruit-quality-related physicochemical parameters, skin coloration, expression levels of important anthocyanin biosynthesis-related structural genes and transcription factors, and total anthocyanin content of ‘Evercrisp’ fruit located in the canopy’s lower third during on-the-tree ripening, for 2 years, under mid-Atlantic US conditions. Fruit treated with reflective groundcovers displayed an enhanced red skin coloration, reaching >60% blush 1 week before commercial harvest and 2 weeks earlier than control fruit. This resulted from a significantly increased transcript accumulation of anthocyanin biosynthesis-assessed genes, which was promoted by an increased light reflectance (>5–25 times greater than control), which also led to a correspondingly higher total anthocyanin content. Additionally, reflective groundcover-treated ‘Evercrisp’ fruit also exhibited an increased IEC and an advanced maturity, but without differences in fruit drop, as compared to control fruit, during on-the-tree ripening. Reflective groundcovers deployment would allow for an earlier harvest (of at least one week) of ‘Evercrisp’ fruit, which would be packing out in the premium grades as compared to control, thus increasing fruit crop value.

## 1 Introduction

Consumer acceptability and increased market value are usually linked to an increased skin blush percentage in apple cultivars (Musacchi and Serra, 2018; Funke and Blanke, 2021). Moreover, currently, a minimum of a 50% blush in apple skin is necessary for fruit packing out in the premium grades. This requisite directly impacts marketability and profitability of important apple cultivars in the mid-Atlantic US, including ‘Evercrisp’ (USDA Agricultural Marketing Service, no date; Kon and Clavet, 2023; Miah and Farcuh, 2024a). ‘Evercrisp’ originated from the Midwestern Apple Improvement Association (MAIA) and is a hybrid of ‘Fuji’ × ‘Honeycrisp’, combining the sweet flavor of ‘Fuji’ with ‘Honeycrisp’ texture attributes (Brown and Maloney, 2013). As a result of the late-ripening nature of ‘Evercrisp’, in the mid-Atlantic US, fruit are frequently harvested before they reach the minimum 50% skin color requirement due to the high risk of freezing events that occur in the region near its optimum commercial harvest period.

Anthocyanin content is a crucial determinant of apple skin blush (Castañeda-Ovando et al., 2009). It has been previously reported that, in apples, anthocyanin content is higher in skin than in flesh tissues (Kunradi Vieira et al., 2009; Łata et al., 2009). Anthocyanins are a result of the phenylpropanoid pathway (Feng et al., 2013). The biosynthesis of anthocyanins begins with phenylalanine, and multiple enzymes are known to be involved in the overall pathway, including phenylalanine ammonia-lyase (PAL), chalcone synthase (CHS), chalcone isomerase (CHI), flavanone 3-hydroxylase (F3H), dihydroflavonol 4-reductase (DFR), leucoanthocyanidin dioxygenase (LDOX), and UDP glucose-flavonoid 3-O-glucosyltransferase (UFGT) (Xie et al., 2011). Transcription factors, such as *MdMYB10*, are known to regulate expression levels of anthocyanin biosynthesis-related structural genes (Ban et al., 2007; Espley et al., 2009; Telias et al., 2011; Ryu et al., 2022), and in apple fruit, transcript accumulation of *MdMYB10* has been shown to rise as anthocyanin content increases (Espley et al., 2007).

Accumulation of anthocyanins predominantly happens throughout apple fruit ripening on the tree (Honda et al., 2002), and it can be significantly impacted by environmental features such as light and temperature (Lancaster, 1992; Ubi et al., 2006; Honda and Moriya, 2018; Miah and Farcuh, 2024a). Specifically, light intensity and wavelength, and, within the latter, ultraviolet (UV) radiation, are crucial factors in controlling anthocyanin content and therefore apple skin blush (Dong et al., 1995; Charles and Arul, 2007; Honda and Moriya, 2018; Toivonen et al., 2019; Chen et al., 2021). In fact, regulatory genes such as the MYB transcription factor and anthocyanin biosynthesis-related structural genes have been shown to be light inducible (Zhiqiang et al., 1999; Kondo et al., 2002; Takos et al., 2006; Ban et al., 2007; Allan et al., 2008; Vimolmangkang et al., 2014; Miah and Farcuh, 2024b). Nevertheless, it is important to consider that light distribution through the tree canopy is generally not uniform, as the outer and upper canopy has been reported to intercept more light than the inner and lower canopy levels (Layne et al., 2002). Additionally, a high proportion of light is also absorbed by the orchard floor instead of reaching the apple fruit surface to stimulate apple red blush development (Wünsche et al., 1996; Robinson and Gonzalez, 2022).

The use of reflective groundcovers is a preharvest management strategy that has been reported to increase canopy light distribution (Layne et al., 2002; Privé et al., 2011). One of the most used reflective groundcovers corresponds to Extenday, a woven white polyethylene fabric, which is highly durable and can be used for more than five seasons (Robinson and Gonzalez, 2022). Reflective groundcovers, such as Extenday, were devised to reflect incoming sunlight back into the canopy, instead of losing it into the orchard floor, boosting light penetration into the inner and lower canopy levels and therefore increasing the light that reaches the fruit (Toye, 1995; Funke and Blanke, 2021; Mupambi et al., 2021; Kon and Clavet, 2023). Generally, reflective groundcovers are deployed between tree rows approximately 4 weeks before commercial harvest and have been shown to enhance apple skin blush in multiple economically important apple cultivars grown in different locations (Layne et al., 2002; Miller and Greene, 2003; Privé et al., 2008; Iglesias and Alegre, 2009; Toivonen et al., 2019; Mupambi et al., 2021; Robinson and Gonzalez, 2022; Kon and Clavet, 2023; Miah and Farcuh, 2024a).

Besides the challenge of reaching the minimum required 50% apple skin blush before freezing events take place in the mid-Atlantic US, ‘Evercrisp’ apples are also susceptible to preharvest fruit drop under our conditions. Preharvest fruit drop can start as early as 3–4 weeks before the commercial harvest of the fruit and thus before the fruit is horticulturally mature (Irish-Brown et al., 2011; Arseneault and Cline, 2018; Liu et al., 2022). Hence, understanding the effect of preharvest strategies, such as the use of reflective groundcovers in fruit drop, is of uttermost importance for increasing the profitability of ‘Evercrisp’ apples in the mid-Atlantic US.

Despite previous studies reporting the positive effects of the use of reflective groundcovers on promoting apple skin blush, integrative studies assessing the impacts of the use of the reflective groundcovers on ‘Evercrisp’ apple preharvest fruit drop, maturity and quality properties, and anthocyanin biosynthesis-related gene expression and total anthocyanin content during ripening on the tree, under the mid-Atlantic environmental conditions, are missing. Considering this background, the goal of the current study was threefold: one, to assess the effect of reflective groundcovers on light interception and reflectance in an ‘Evercrisp’ orchard located in the mid-Atlantic US; two, to characterize and compare changes in preharvest fruit drop, internal ethylene concentration (IEC), fruit-quality-related physicochemical parameters, skin coloration and expression levels of important anthocyanin biosynthesis-related structural genes and transcription factors, and total anthocyanin content of ‘Evercrisp’ fruit subjected to reflective groundcover deployment during on-the-tree ripening; and three, to find significant correlations among the evaluated features using multivariate analysis.

## 2 Materials and methods

### 2.1 Tree fruit and reflective groundcover deployment

This work was conducted in a mature commercial ‘Evercrisp’/’G.41’ apple block in Aspers, PA (39.96° N, 77.28° W). The apple block is located in a humid continental climate with an annual average precipitation of 800 mm and a minimum and maximum relative humidity average ranging between 44% to 82%, respectively, throughout the year. Average daily minimum and maximum temperatures throughout the year range between 8.5°C and 18.2°C, respectively, with freezing events occurring from November through March/April. Trees were trained to a tall spindle system and planted at 1 × 4 m spacing. In both seasons (2021 and 2022), 4 weeks before the projected commercial harvest date, the reflective groundcover Extenday (Extenday New Zealand, Auckland, New Zealand), comprised of a white woven polyethylene reflective groundcover with a 3.5-m width, was placed contiguous to 30-tree plots on each side of the row and secured as indicated by the fabricator. Control plots were also included for comparison. Within the row, reflective groundcover-treated and control plots were separated by minimum 20 trees, while a minimum of three-tree rows on each side were used to isolate treatments. The study used a randomized complete block design with four replications.

Each year, fruit maturity indices [i.e., surface and background skin coloration, skin blush, flesh firmness, starch pattern index (SPI), soluble solids contents (SSC) and titratable acidity (TA)] were assessed in ‘Evercrisp’ fruit across the season (Miah and Farcuh, 2024a). Evaluation periods comprised three on-the-tree ripening stages: 1 week before optimal commercial harvest (1WBCH), optimal commercial harvest (CH), and 1 week after CH (CH + 1W). At each evaluation period, for each of the four replications of each treatment, 25 fruit were collected from the canopy’s lower third (1.5 m above the ground) internal section. Per replication, internal ethylene concentration (IEC) was analyzed in five fruit, and these same fruit were also rinsed, and the skin was removed and pooled together, subjected to liquid nitrogen, and stored at −80°C for downstream examination, while the other 20 fruit were used for evaluation of the physicochemical properties and skin coloration.

### 2.2 Quantification of light interception and reflectance

Quantification of light interception and reflectance in Extenday reflective groundcover and control treatments was performed in the mid-row (middle of the drive row) and in-canopy (within the canopy) on a cloudy (18 October 2021 and 17 October 2022) and sunny (20 October 2021 and 21 October 2022) day each year. In all measurement dates, the fruit ripening stage was between 1WBCH and CH. In general, for each replication, measurements were executed 1.5 m above the ground, on two mid-row locations and on two trees in the middle of each 30-tree-plot replication. Following what was previously described (Miah and Farcuh, 2024a), light interception was quantified with sensors positioned in the direction of the sun, while light reflectance was determined by inverting the sensors (in the direction of the reflective groundcover). A LI-COR LI-191R Line Quantum Sensor attached to an LI-250A Light Meter (LI-COR Environmental, Lincoln, NE, US) was used to assess photosynthetic photon flux density (PPFD; 400–700 nm waveband; µmol m^−2^ s^−1^). Two measurements of light interception and reflectance were obtained, with the sensor located perpendicular to the row, once each on the north and south side of the trunk. For in-canopy measurements, the distal end of the sensor was positioned next to the trunk. Quantification of ultraviolet light (UV; 250–400 nm; µmol m^−2^ s^−1^) was achieved using a portable UV meter (FieldScout model 3414F, Spectrum Technologies Inc., Aurora, IL, US). For each tree, four measurements of interception and reflectance were obtained, once at each of the four points around the trunk (south, west, north, and east). For in-canopy measurements, the UV meter was located 15 cm from the trunk.

### 2.3 Fruit drop evaluations

Fruit drop evaluations were performed as described before (Miah and Farcuh, 2024b). Three weeks before CH, for each replication within the Extenday reflective groundcover and control treatments, five limbs, selected from different trees and comprising 20 fruit each, were marked. Fruit drop evaluations were conducted by weekly assessment of the marked fruit that persisted on the limb from 2WBCH through CH +1W. Fruit drop was a calculated as percentage in relation to the initial fruit number per limb.

### 2.4 Fruit internal ethylene concentration

Fruit internal ethylene concentration (IEC) was assessed via extraction of 1-mL samples of internal gas from the core cavity of each evaluated fruit using a gas chromatograph (GC-2014C, Shimadzu Co., Kyoto, Japan) as previously described (Farcuh and Hopfer, 2023; Miah et al., 2023; Miah and Farcuh, 2024b).

### 2.5 Fruit physicochemical properties and skin coloration measurements

Evaluation of fruit weight, surface and background skin coloration, index of absorbance difference (I_AD_), skin red blush percentage, flesh firmness, starch pattern index (SPI), soluble solids contents (SSC), and titratable acidity (TA) was conducted as described before (Miah et al., 2023; Miah and Farcuh, 2024a). In general, the weight of the fruit was measured via an electronic balance (Sartorius, AG Gottingen, Germany). Surface and background skin coloration was evaluated using a colorimeter (Konica Minolta CR400 Chroma Meter, Konica Minolta Sensing, Inc., Osaka, Japan) on two opposed sides of each fruit along the equatorial axes, and hue angle (hue°) was calculated based on previous reports (Infante et al., 2008). Additionally, the index of absorbance difference (I_AD_) was quantified using a DA-Meter (TR Turoni, Forli, Italy) by measuring at three different positions on each fruit (Ziosi et al., 2008). Flesh fruit firmness, expressed in N, was evaluated via a TA.XT Plus Connect texture analyzer (Texture Technologies Corp., Scarsdale, NY, US) equipped with a 50-kg load cell and analyzed with the Exponent TE32 (v6.0, Texture Technologies Corp., Scarsdale, NY, US) software fitted with an 11.1-mm diameter probe, in two opposed sides of each fruit with the skin removed. SPI values were obtained using the Cornell generic chart (Blanpied and Silsby, 1992). SSC and TA were assessed via a digital hand-held refractometer (Atago, Tokyo, Japan) and automatic titrator (855 Robotic Titrosampler; Metrohm, Riverview, FL, US), respectively (Farcuh et al., 2018, 2020).

### 2.6 Real-time quantitative RT-PCR analysis

RNA isolation from apple skin was performed via the cetyltrimethylammonium bromide (CTAB)/NaCl method (Chang et al., 1993), with some variations as previously described (Kim et al., 2015; Farcuh et al., 2018; Miah and Farcuh, 2024b). First-strand complementary DNA synthesis, primer design, and quantitative PCR were conducted following previous studies (Kim et al., 2015). Primer sequences used in this work are found in **Supplementary Table S1**. Relative gene expression analysis was based on the comparative cycle threshold method (Livak and Schmittgen, 2001) using actin (*MdACT*) as a reference gene.

### 2.7 Total anthocyanin content

Total anthocyanin content in apple skin was performed, recording absorbance at 530 nm using a Cary 60 UV–Vis (Agilent Technologies, Palo Alto, CA, US) spectrophotometer, based on the method previously described (Whale and Singh, 2007; Miah and Farcuh, 2024b). Total anthocyanin content was obtained via molar extinction coefficient (i.e., 3.43 × 10^4^) for idaein chloride (Siegelman and Hendricks, 1958).

### 2.8 Statistical analysis

Generalized linear mixed models were used to model the response variables including treatments and evaluation periods (ripening stages) as fixed factors and block as a random factor to determine the statistical significance of the interactions and main effects (analysis of variance, ANOVA). If statistically significant differences were obtained, separation of means was achieved via Tukey’s HSD test at a significance level of 5%. Main effects were assessed only when there was no significant interaction detected. The data met normality and homogeneity of variance assumptions.

For each pairwise combination of evaluated features, Pearson’s correlation coefficients, using mean-centered data, were estimated. A “biplot” graph was used to visualize the PCA, denoting the relationships among the variables (fruit drop, IEC, physicochemical properties, skin coloration, gene expression values, and anthocyanin contents) and the assessed treatments and evaluation periods (ripening stages). In order to define the number of principal components that capture most of the variation, the Scree test was employed, defined by plot of the magnitude of an eigenvalue (= the variance of the principal component) versus its number. All the statistical analyses were performed using the software package JMP (ver 15.2, SAS Institute, Cary, NC, US).

## 3 Results

### 3.1 Reflective groundcover effect on the quantification of light interception and reflectance

Intercepted PPFD and UV radiation (µmol m^−2^ s^−1^) were significantly higher in sunny as compared to cloudy days for both mid-row and in-canopy but displayed no differences between reflective groundcover and control, consistently during both years (**Tables 1**, **2**). On the other hand, reflected PPFD and UV radiation (µmol m^−2^ s^−1^) displayed significant differences when comparing cloudy and sunny days and between treatments, for both mid-row and in-canopy. The highest PPFD and UV reflectance values (µmol m^−2^ s^−1^) were observed for reflective groundcover treatment evaluated on sunny days, followed by reflective groundcover treatment assessed on cloudy days, which presented significantly higher values than the control, consistently during both years. The control treatment measured on cloudy days displayed the significantly lowest PPFD and UV reflectance values (µmol m^−2^ s^−1^) (**Tables 1**, **2**). In general, including sunny and cloudy days and the 2 years of study, PPFD and UV reflectance (µmol m^−2^ s^−1^) from reflective groundcovers measured in the mid-row displayed values that were 5–20 times larger than control, and PPFD and UV reflectance from reflective groundcovers measured in-canopy exhibited values that were 5–25 times larger than control (**Tables 1**, **2**).

**Table 1:** Reflective groundcover (Extenday) effect on PPFD and UV radiation interception and reflection in the mid-row (middle of the drive row) and in-canopy (within canopy) of ‘Evercrisp’ trees on a sunny day and on a cloudy day in Aspers, PA in 2021.

PPFD (400–700 nm; µmol m^−2^ s^−1^)								
	Mid-row				In- canopy^z^			
	Intercepted^y^		Reflected^x^		Intercepted		Reflected	
Treatment	Cloudy day	Sunny day	Cloudy day	Sunny day	Cloudy day	Sunny day	Cloudy day	Sunny day
Extenday	363±24.1 b	926±19.5 a	151±14.5 b	415±5.8 a	148±11.6 b	330±15.3 a	50 ±4.3 b	106±10.4 a
Control	347±15.4 b	909±28.4 a	15±1.1 d	54±5.2 c	139±9.3 b	321±12.1 a	2±0.2 d	20±1.2 c
UV (250-400 nm; µmol m^−2^ s^−1^)								
	Mid-row				In- canopy ^w^			
	Intercepted		Reflected		Intercepted		Reflected	
Treatment	Cloudy day	Sunny day	Cloudy day	Sunny day	Cloudy day	Sunny day	Cloudy day	Sunny day
Extenday	46±3.7 b	97±7.3 a	20±1.4 b	38±2.8 a	10±1.2 b	26±1.3 a	7±0.7 b	14±1.9 a
Control	39±2.3 b	103±9.4 a	2±0.1 d	8±0.2 c	7±0.4 b	24±1.0 a	0.3±0.1 d	2±0.1 c

PPFD, photosynthetic photon flux density; UV, ultraviolet radiation. ^z^In-canopy PPFD measurements were carried out with the distal end of the sensor next to the trunk. ^y^Intercepted light was determined with sensors oriented towards the sun (sky) approximately 1.5 m above the ground. ^x^Reflected light was quantified by inverting the sensors (facing the orchard floor or the reflective groundcover). ^w^In-canopy UV measurements were performed with the UV meter positioned 15 cm from the trunk. Values are means ± standard error. Different letters indicate significant differences (p ≤ 0.05) between both treatments and cloudy/sunny conditions, within intercepted and reflected values in each case, according to Tukey’s HSD test.

**Table 2:** Reflective groundcover (Extenday) effect on PPFD and UV radiation interception and reflection in the mid-row (middle of the drive row) and in-canopy (within canopy) of ‘Evercrisp’ trees on a sunny day and on a cloudy day in Aspers, PA in 2022.

PPFD (400–700 nm; µmol m^−2^ s^−1^)								
	Mid-row				In-canopy^z^			
	Intercepted^y^		Reflected^x^		Intercepted		Reflected	
Treatments^w^	Cloudy day	Sunny day	Cloudy day	Sunny day	Cloudy day	Sunny day	Cloudy day	Sunny day
Extenday	584±17.6 b	1158±25.2 a	387±10.4 b	681±15.1 a	280±13.4 b	413±18.2 a	127±7.5 b	173±14.2 a
Control	572±14.2 b	1142±21.1 a	20±1.9 d	85±10.4 c	268±11.7 b	405±11.4 a	5±0.5 d	31±2.1 c
UV (250-400 nm; µmol m^−2^ s^−1^)								
	Mid-row				In-canopy^w^			
	Intercepted		Reflected		Intercepted		Reflected	
Treatments	Cloudy day	Sunny day	Cloudy day	Sunny day	Cloudy day	Sunny day	Cloudy day	Sunny day
Extenday	58±2.9 b	189±16.5 a	37±2.4 b	60±5.4 a	13±1.7 b	31±5.4 a	11±1.9 b	21±2.0 a
Control	52±3.7 b	177±15.3 a	3±0.6 d	13±1.8 c	11±1.1 b	28±3.1 a	0.8±0.1 d	4±0.3 c

PPFD, photosynthetic photon flux density; UV, ultraviolet radiation. ^z^In-canopy PPFD measurements were carried out with the distal end of the sensor next to the trunk. ^y^Intercepted light was determined with sensors oriented towards the sun (sky) approximately 1.5 m above the ground. ^x^Reflected light was quantified by inverting the sensors (facing the orchard floor or the reflective groundcover). ^w^In-canopy UV measurements were performed with the UV meter positioned 15 cm from the trunk. Values are means ± standard error. Different letters indicate significant differences (p ≤ 0.05) between both treatments and cloudy/sunny conditions, within intercepted and reflected values in each case, according to Tukey’s HSD test.

### 3.2 Reflective groundcover effect on the ‘Evercrisp’ fruit drop percentage throughout ripening on the tree

**Figure 1:**
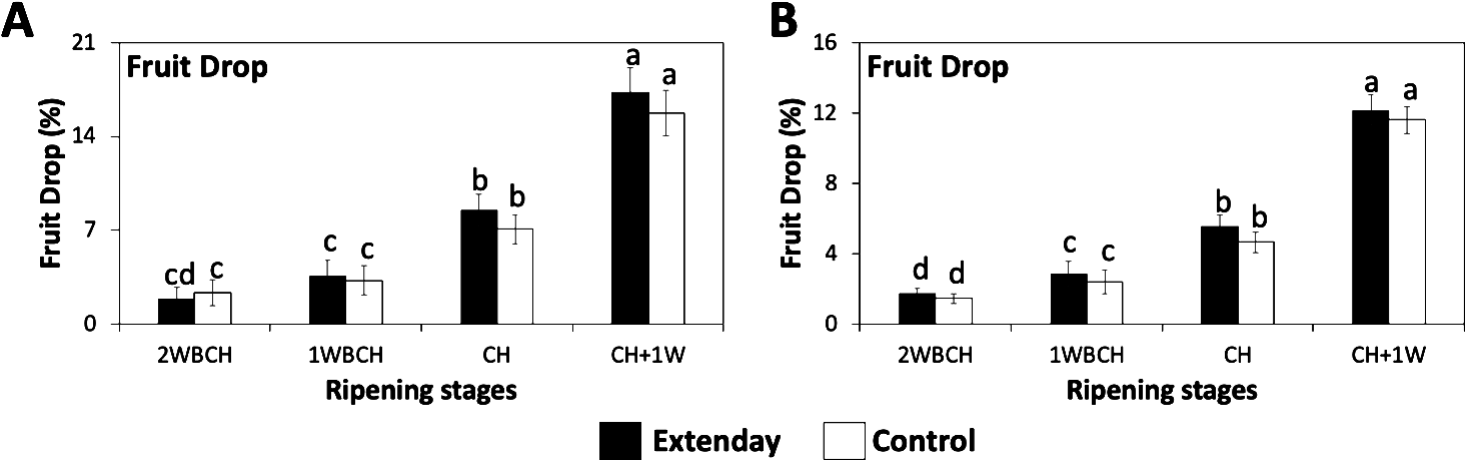
Reflective groundcover (Extenday) effect on ‘Evercrisp’ preharvest fruit drop in Aspers, PA. Preharvest fruit drop was evaluated in **(A)** 2021 and **(B)** 2022. Apples were assessed 2 weeks before optimal commercial harvest (2WBCH), 1 week before optimal commercial harvest (1WBCH), at optimal commercial harvest (CH), and 1 week after CH (CH + 1W). Values are means ± standard error. Different letters indicate significant differences (p ≤ 0.05) between treatments and ripening stages, for each assessed parameter, according to Tukey’s HSD test.

Fruit drop percentage displayed a statistically significant increase throughout ripening on the tree, i.e., from 2WBCH to CH + 1W, for both treatments, consistently in both years of the study (**Figure 1**). In both treatments, at CH + 1W, fruit drop percentage reached ~17% in 2021 and ~12% in 2022. In 2021, there were no significant differences between 2WBCH and 1WBCH, but in 2022, the differences between these two ripening stages were significant, with the former presenting significantly lower fruit drop percentage values than the latter. Regarding treatments, there were no differences between reflective groundcover-treated and control fruit drop percentage in any of the assessed evaluation periods in 2021 or 2022 (**Figure 1**).

### 3.3 Reflective groundcover effect on internal ethylene concentration and physicochemical properties of ‘Evercrisp’ fruit throughout ripening on-the-tree

**Figure 2:**
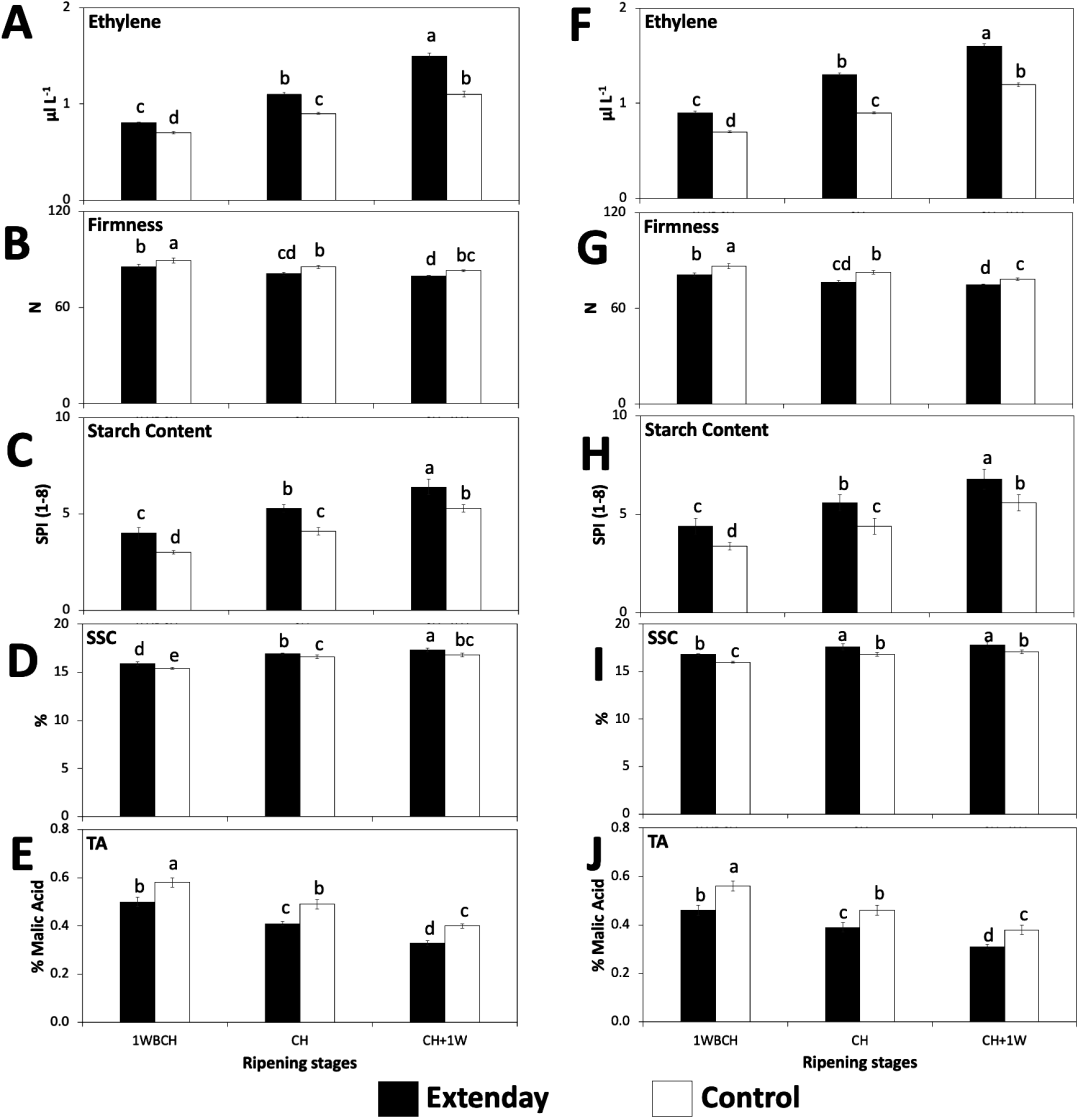
Reflective groundcover (Extenday) effects on internal ethylene concentration and physicochemical properties of ‘Evercrisp’ apples collected throughout ripening on the tree in Aspers, PA in **(A–E)** 2021 and **(F–J)** 2022. **(A, F)** Internal ethylene concentration, **(B, G)** flesh firmness, **(C, H)** starch content (starch pattern index), **(D, I)** soluble solids contents, and **(E, J)** titratable acidity. Apples were assessed 1 week before optimal commercial harvest (1WBCH), at optimal commercial harvest (CH), and 1 week after CH (CH + 1W). N, Newton; SPI, starch pattern index (1−8 scale); SSC, soluble solids contents; TA, titratable acidity. Values are means ± standard error. Different letters indicate significant differences (p ≤ 0.05) between treatments and ripening stages, for each assessed parameter, according to Tukey’s HSD test.

Internal ethylene concentration (μL L^-1^) exhibited a significant increase throughout ripening on-the-tree for reflective groundcover-treated and control fruit in both years of the study, with CH +1W presenting the highest internal ethylene concentration (IEC) for each treatment (**Figures 2A, F**). For each assessed ripening stage, reflective groundcover treatment consistently displayed a significantly higher IEC (μL L^−1^) than control fruit in both assayed years. The significantly highest IEC values (μL L^−1^) were exhibited by reflective groundcover-treated fruit at CH + 1W, followed by reflective groundcover-treated and control fruit at CH and CH + 1W, respectively, subsequently by reflective groundcover-treated and control fruit at 1WBCH and CH, respectively, whereas the significantly lowest IEC values were observed for control fruit at 1WBCH (**Figures 2A, F**).

Fruit weight (g) did not present significant differences throughout the assessed ripening stages nor between treatments in any of the 2 years evaluated in this work (data not shown).

Values of fruit flesh firmness (N), in both years and for both treatments, decreased as ‘Evercrisp’ apples ripening stages advanced on the tree, although there were no differences observed between CH and CH + 1W in reflective groundcover-treated fruit in 2021 and 2022 or in control-treated fruit in 2021 (**Figures 2B, G**). When comparing between treatments, flesh firmness values (N) were significantly lower in reflective groundcover-treated fruit as compared to control at all evaluated periods in both years. Particularly, at CH + 1W, reflective groundcover-treated fruit displayed values <80N and <75N in 2021 and 2022, respectively, while control fruit presented values >80N and >75N correspondingly (**Figures 2B, G**).

Starch pattern index was also affected by evaluation periods and by treatments (**Figures 2C, H**). In 2021 and 2022, SPI values, on a scale from 1 to 8, significantly increased (demonstrating a decrease in starch content) in both treatments throughout ripening on the tree. Furthermore, within treatments, in both years, reflective groundcover-treated fruit exhibited higher SPI values at all stages, as compared to control fruit (**Figures 2C, H**). In general, the significantly highest SPI values, on a scale from 1 to 8, were observed for reflective groundcover-treated fruit at CH + 1W (>6.0), followed by reflective groundcover-treated and control fruit at CH and CH + 1W, respectively (5.3–5.6), which were significantly different than reflective groundcover-treated and control fruit at 1WBCH and CH, respectively (4.0–4.4), while the lowest SPI values were observed for control fruit at 1WBCH (<3.5) (**Figures 2C, H**).

Soluble solid contents (%) showed a trend to rise in their values throughout the three assayed evaluation periods for both treatments, although no significant differences were observed between CH and CH + 1W in reflective groundcover-treated fruit in 2022 and in control fruit in 2021 and 2022 (**Figures 2D, I**). Within treatments, SSC values (%) were significantly higher in reflective groundcover-treated fruit as compared to control at all evaluated periods, consistently in both years. At CH + 1W, reflective groundcover-treated fruit displayed values ~17.5%, while control fruit had SSC ~17%, in average considering both years (**Figures 2D, I**).

Titratable acidity (% malic acid) displayed a significant reduction throughout ripening on the tree for both treatments for the two assayed years (**Figures 2E, J**). Additionally, in 2021 and 2022, reflective groundcover-treated fruit exhibited lower TA values at all stages, as compared to control fruit. In fact, the significantly highest TA values (% malic acid) were observed for control fruit at 1WBCH (>0.55), followed by control fruit and reflective groundcover-treated fruit at CH and 1WBCH, respectively (0.46–0.50), which were significantly different than control fruit and reflective groundcover-treated fruit at CH + 1W and CH, respectively (0.38–0.40), while the lowest TA values were observed for reflective groundcover-treated fruit at CH + 1W (<0.35) (**Figures 2E, J**).

### 3.4 Reflective groundcover effect on skin coloration of ‘Evercrisp’ fruit throughout ripening on the tree

**Figure 3:**
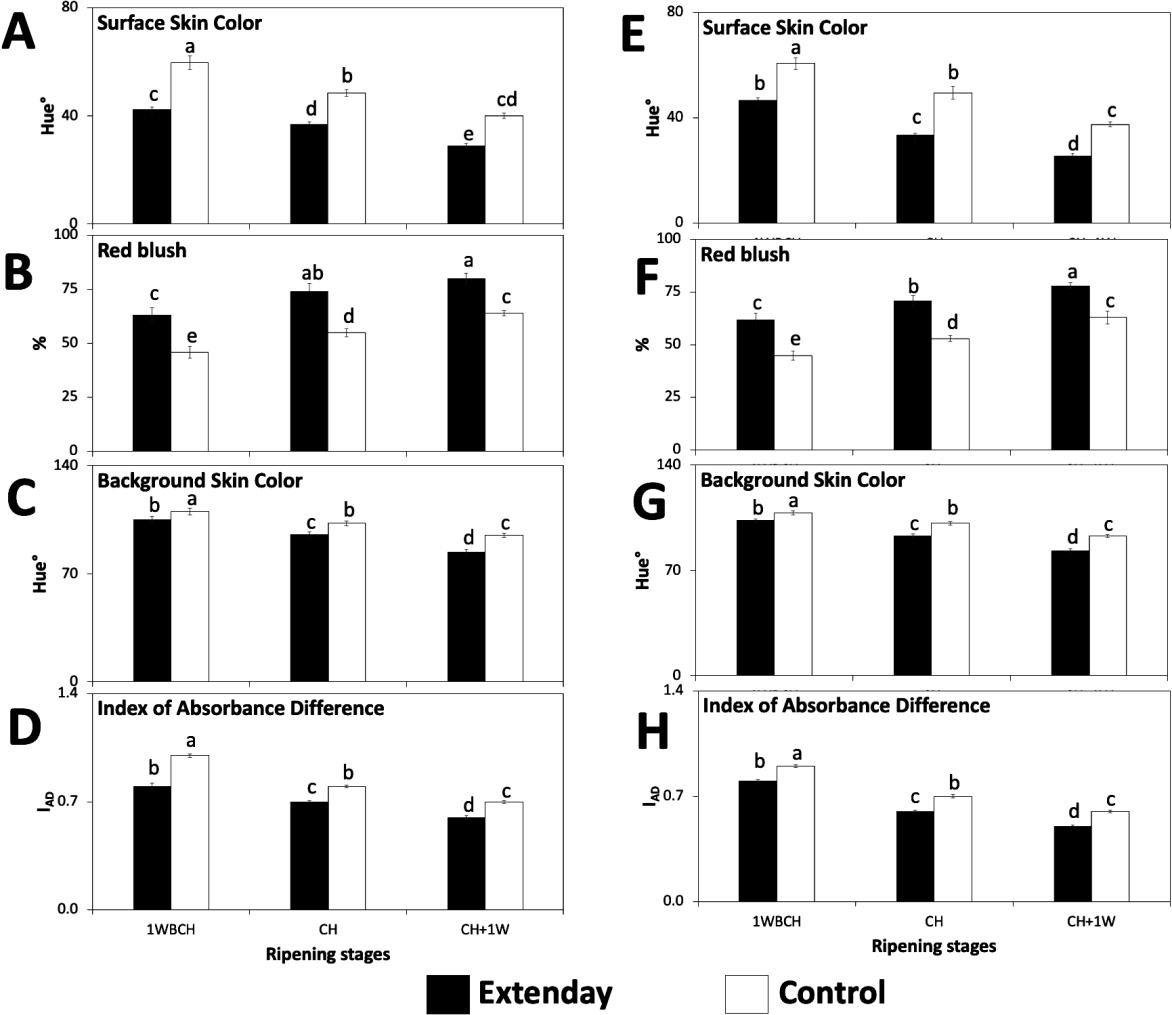
Reflective groundcover (Extenday) effects on surface and background skin coloration of ‘Evercrisp’ apples collected throughout ripening on the tree in Aspers, PA in **(A–D)** 2021 and **(E–H)** 2022. **(A, E)** Surface skin color, **(B, F)** red blush, **(C, G)** background skin color, and **(D, H)** index of absorbance difference. Apples were assessed 1 week before optimal commercial harvest (1WBCH), at optimal commercial harvest (CH), and 1 week after CH (CH + 1W). Values are means ± standard error. Different letters indicate significant differences (p ≤ 0.05) between treatments and ripening stages, for each assessed parameter, according to Tukey’s HSD test.

Surface skin hue angle values (°) exhibited a significant decrease (i.e., revealing an increase in red skin coloration) as the fruit ripened on the tree in both treatments, for 2021 and 2022, with fruit assayed at CH +1W presenting the lowest skin hue angle values for each treatment (**Figures 3A, E**). For each assessed ripening stage, reflective groundcover treatment consistently displayed fruit with significantly lower skin hue angle values (°) than control in both years (**Figures 3A, E**). Consistent with the resulting skin hue angle values, the evaluation of red blush percentage presented a significant increase from 1WBCH to CH + 1W for reflective groundcover-treated and control fruit in both years (**Figures 3B, F**), although in 2021, no significant differences were observed between CH and CH + 1W in reflective groundcover-treated fruit (**Figure 3B**). Additionally, in both years, when comparing between treatments, the significantly highest skin blush percentage values were observed for reflective groundcover-treated fruit at all stages throughout ripening on the tree (**Figures 3B, F**). In fact, reflective groundcover-treated fruit reached >60% red blush in fruit harvested at 1WBCH, while control fruit only achieved these values in the last evaluation period, i.e., when harvested at CH + 1W (63%–64%) (**Figures 3B, F**).

Background skin color hue angle (°) (**Figures 3C, G**) together with the assessment of index of absorbance difference (I_AD_) (**Figures 3D, H**) showed a significant reduction in their values (demonstrating a change in coloration from green to yellow, and a reduction in chlorophyll content, respectively) from 1WBCH through CH + 1W in both reflective groundcover-treated and control ‘Evercrisp’ fruit, in 2021 and 2022. Furthermore, in both years, for each evaluated ripening stage, reflective groundcover-treated fruit displayed significantly lower background skin hue angle (°) and I_AD_ values than control fruit (**Figures 3C, D, G, H**). The significantly highest background skin hue angle (°) and I_AD_ values were observed for control fruit at 1WBCH, followed by control fruit and reflective groundcover-treated fruit at CH and 1WBCH, respectively, subsequently by control fruit and reflective groundcover-treated fruit at CH +1W and CH, respectively, while the lowest background skin hue angle and I_AD_ values were observed for reflective groundcover-treated fruit at CH + 1W (**Figures 3C, D, G, H**).

### 3.5 Reflective groundcover effect on the expression of key anthocyanin biosynthesis-related genes in ‘Evercrisp’ fruit skin throughout ripening on the tree

**Figure 4:**
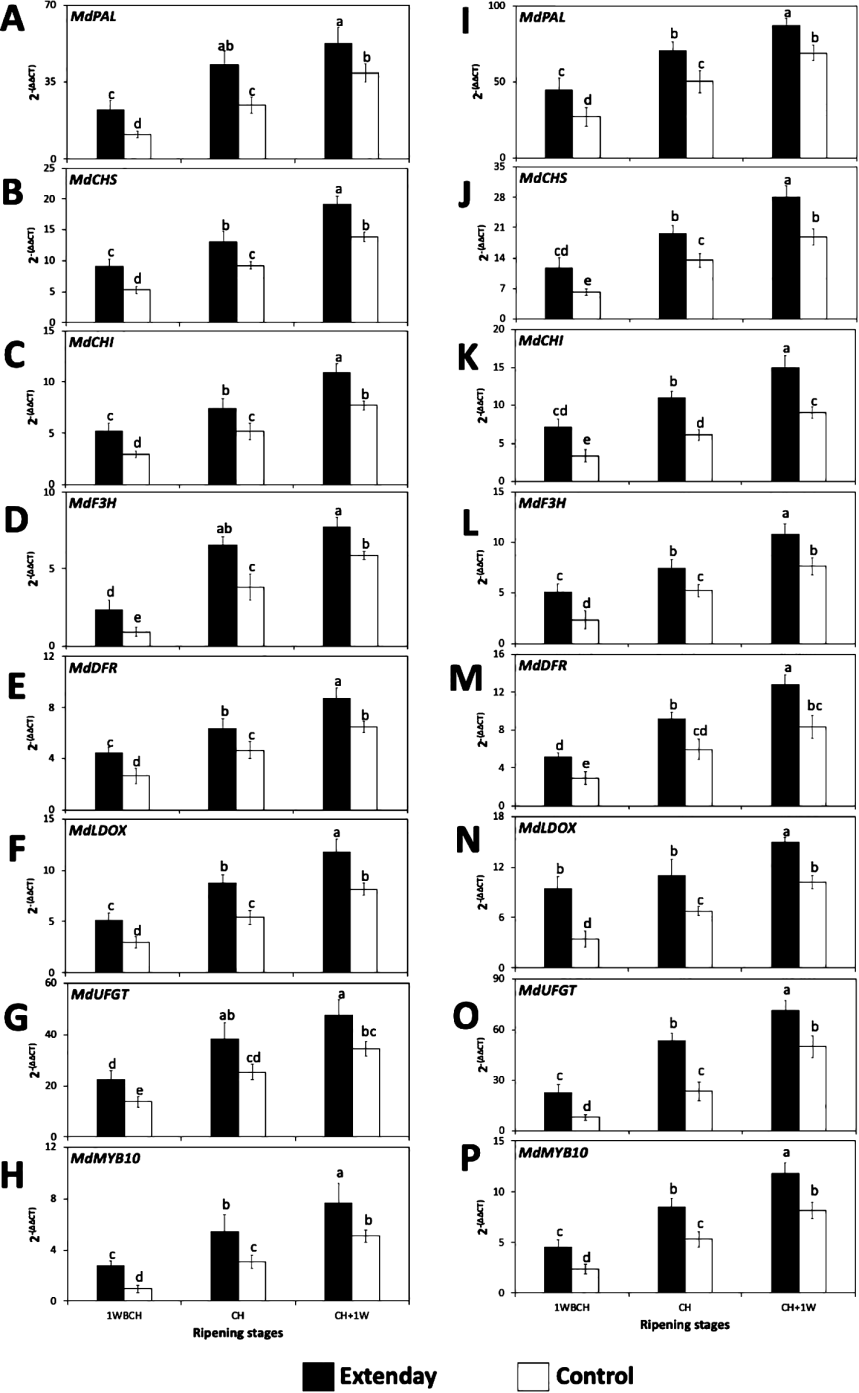
Reflective groundcover (Extenday) effects on the expression levels of anthocyanin biosynthesis-related genes of ‘Evercrisp’ apples from Aspers, PA in **(A–H)** 2021 and **(I–P)** 2022. **(A, I)** *MdPAL*, **(B, J)** *MdCHS*, **(C, K)** *MdCHI*, **(D, L)** *MdF3H*, **(E, M)** *MdDFR*, **(F, N)** *MdLDOX*, **(G, O)**, *MdUFGT*, and **(H, P)** *MdMYB10*. Apples were assessed 1 week before optimal commercial harvest (1WBCH), at optimal commercial harvest (CH), and 1 week after CH (CH + 1W). Values are means ± standard error. Different letters indicate significant differences (p ≤ 0.05) between treatments and ripening stages, for each assessed parameter, according to Tukey’s HSD test. Phenylalanine ammonia-lyase (PAL), chalcone synthase (CHS), chalcone isomerase (CHI), flavanone 3-hydroxylase (F3H), dihydroflavonol 4-reductase (DFR), leucoanthocyanidin dioxygenase (LDOX), and UDP glucose-flavonoid 3-O-glucosyltransferase (UFGT).

Evaluation of the transcript accumulation of seven important anthocyanin biosynthesis-related structural genes (*MdPAL*, *MdCHS*, *MdCHI*, *MdF3H*, *MdDFR*, *MdLDOX*, and *MdUFGT*) and one major transcription factor (*MdMYB10*) was conducted in reflective groundcover-treated and control ‘Evercrisp’ apples in 2021 and 2022 (**Figure 4**).

Consistently in both years, all assessed anthocyanin-related structural genes and the transcription factor exhibited a significant upregulation as fruit ripened on the tree, i.e., from 1WBCH to CH + 1W in both treatments (**Figure 4**). In 2021, no significant differences were observed in gene expression between CH and CH + 1W in reflective groundcover-treated fruit for *MdPAL* and *MdF3H*, while for *MdUFGT*, this situation was observed for both treatments (**Figures 4A, D, G**). In 2022, a lack of significant differences were observed in gene expression between CH and CH + 1W in control fruit for *MdDFR* and between 1WBCH and CH in reflective groundcover-treated fruit for *MdLDOX* (**Figures 4M, N**). Moreover, in 2021 and 2022, within each evaluation period, reflective groundcover-treated fruit always displayed significantly higher gene expression in all anthocyanin-related genes as compared to control fruit. In most cases, the significantly highest gene expression values were observed for reflective groundcover-treated fruit at CH + 1W, followed by reflective groundcover-treated and control fruit at CH and CH + 1W, respectively, subsequently by reflective groundcover-treated and control fruit at 1WBCH and CH, respectively, while the lowest gene expression values were observed for control fruit at 1WBCH (**Figure 4**).

### 3.6 Reflective groundcover effect on total anthocyanin content of ‘Evercrisp’ fruit skin throughout ripening on the tree

**Figure 5:**
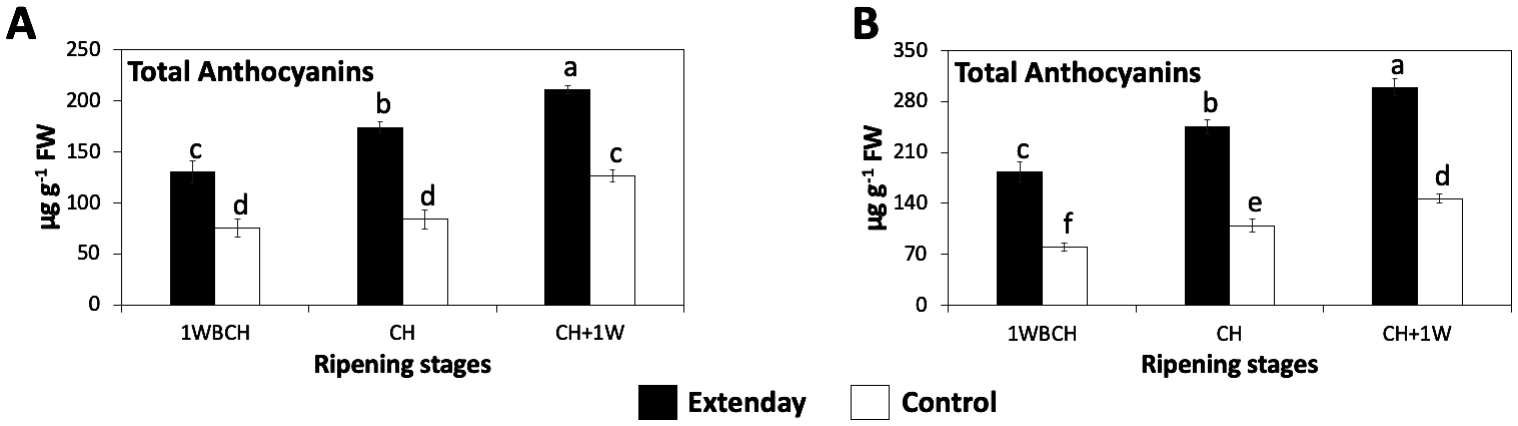
Reflective groundcover (Extenday) effects on total anthocyanin content of ‘Evercrisp’ apples from Aspers, PA in **(A)** 2021 and **(B)** 2022. Apples were assessed 1 week before optimal commercial harvest (1WBCH), at optimal commercial harvest (CH), and 1 week after CH (CH + 1W). Values are means ± standard error. Different letters indicate significant differences (p ≤ 0.05) between treatments and ripening stages, for each assessed parameter, according to Tukey’s HSD test.

A significant rise in total anthocyanin content (µg g^−1^ FW) throughout ripening on the tree was displayed by fruit subjected to reflective groundcover deployment and control fruit, resulting in a 1.5-to 1.8-fold increase from 1WBCH to CH + 1W, including both assayed years (**Figure 5**). Lack of significant differences was only observed for control fruit between 1WBCH and CH in 2021 (**Figure 5A**). At every evaluation period, in both years, reflective groundcover-treated ‘Evercrisp’ apples always presented a significantly higher total anthocyanin content (ranging between 130 µg g^−1^ FW and 300 µg g^−1^ FW) with respect to control fruit (ranging between 75 µg g^−1^ FW and 146 µg g^−1^ FW) (**Figure 5**).

### 3.7 Associations between fruit drop, IEC, physicochemical properties, skin coloration, expression of key anthocyanin biosynthesis-related genes, and total anthocyanin content in ‘Evercrisp’ fruit subjected to reflective groundcovers throughout ripening on the tree

**Figure 6:**
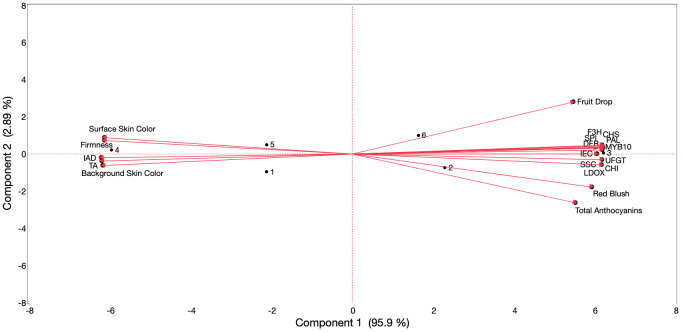
Principal component analysis of results obtained from internal ethylene concentration (IEC), physicochemical parameters, skin coloration, key anthocyanin biosynthesis-related genes, and total anthocyanin content of ‘Evercrisp’ apples subjected to reflective groundcover treatment throughout ripening on the tree. Numbers correspond to the different treatments and evaluation periods that were examined [1 (reflective groundcover Extenday_1WBCH), 2 (reflective groundcover Extenday_CH), 3 (reflective groundcover Extenday_CH + 1W), 4 (Control_1WBCH), 5 (Control_CH), and 6 (Control_CH + 1W)]. Codes for genes are defined in **Figure 4**.

Considering all the assessed features in this study for ‘Evercrisp’ fruit throughout the different ripening stages on the tree, Pearson correlation coefficients were estimated (**Supplementary Table S2**) and a principal component analysis (PCA) was conducted (**Figure 6**), including both years.

Fruit drop exhibited a significant and positive correlation with IEC (r = 0.91), SPI (r = 0.90), SSC (r = 0.82), and skin blush (r = 0.72), with all the anthocyanin biosynthetic-related structural genes (r ≥ 0.84), *MdMYB10* (r = 0.90), and with total anthocyanin content (r = 0.61). On the other hand, fruit drop displayed a significantly negative correlation with fruit flesh firmness (r = −0.80), TA (r = −0.90), surface and background skin hue angles (r = −0.80 and r = −0.92, respectively), and I_AD_ (r = −0.88).

IEC presented a significant and positive association with SPI (r = 0.98), SSC (r = 0.95), skin blush (r = 0.93), and with all the anthocyanin biosynthesis-related structural genes (r ≥ 0.95), *MdMYB10* (r = 0.98), and total anthocyanin content (r = 0.87) (**Supplementary Table S2**), but it showed a negative correlation with flesh firmness (r = −0.93), TA (r = −0.95), surface and background skin hue angles (r = −0.92 and r = −0.97, respectively), and I_AD_ (r = −0.96).

Regarding fruit flesh firmness, this feature significantly and positively correlated with TA (r = 0.95), surface and background skin hue angles (r = 0.96 and r = 0.93, respectively), and I_AD_ (r = 0.97), while it negatively associated with SPI (r = −0.96), SSC (r = −0.96), skin blush (r = −0.95), anthocyanin biosynthesis-related structural genes (r ≤ −0.94), *MdMYB10* (r = −0.95), and total anthocyanin content (r = −0.88) (**Supplementary Table S2**).

SPI positively correlated with SSC (r = 0.97), skin blush (r = 0.90), all anthocyanin biosynthetic-related structural genes (r ≥ 0.95), *MdMYB10* (r = 0.96), and total anthocyanin content (r = 0.84); however, SPI negatively associated with TA (r = −0.94), surface and background skin hue angles (r = −0.95 and r = −0.95, respectively), and I_AD_ (r = −0.93) (**Supplementary Table S2**). SSC showed positive associations with skin blush (r = 0.91), with all the anthocyanin biosynthesis-related structural genes (r ≥ 0.92), *MdMYB10* (r = 0.94), and total anthocyanin content (r = 0.81), while SSC negatively associated with TA (−0.93), surface and background skin hue angles (r = −0.94 and r = −0.92, respectively), and I_AD_ (r = −0.95). Conversely, TA positively correlated with surface and background skin hue angles (r = 0.96 and r = 0.95, respectively) and I_AD_ (r = 0.97), but presented significantly negative associations with fruit skin blush (r = −0.90), with all anthocyanin biosynthetic-related structural genes (r ≤ −0.92), *MdMYB10* (r = −0.95), and with total anthocyanin content (r = −0.81).

Considering all color-related features, surface and background skin hue angles and I_AD_ were all significantly and positively associated among them (r ≥ 0.95) and negatively correlated with skin blush (r ≤ −0.91), with all anthocyanin biosynthesis-related structural genes (r ≤ −0.93), *MdMYB10* (r ≤ −0.94), and with total anthocyanin content (r ≤ −0.82) (**Supplementary Table S2**). On the other hand, skin blush exhibited significantly positive correlations with all assayed anthocyanin-related genes (r ≥ 0.89) and with total anthocyanin content (r = 0.93).

All anthocyanin biosynthesis-related structural genes positively correlated among them (r ≥ 0.94), with *MdMYB10* (r ≥ 0.95), and with total anthocyanin content (r ≥ 0.92). Furthermore, *MdMYB10* significantly and positively associated with total anthocyanin content (r = 0.85) (**Supplementary Table S2**).

PCA results show that the two first principal components explained 95.9% (component 1) and 2.89% (component 2) of the total variation (98.79%) (**Figure 6**). The positioning of the different combinations of treatments and evaluation periods alongside component 1 was determined by surface and background skin hue angle, I_AD_, flesh firmness, and TA for the negative side of the axis (and associated with control fruit at 1WBCH and CH and reflective groundcover-treated fruit at 1WBCH), and by fruit drop, IEC, SPI, SSC, skin blush, all the evaluated anthocyanin biosynthesis-related structural genes, *MdMYB10*, and total anthocyanin content for the positive side of the axis (and associated with control fruit at CH + 1W and reflective groundcover- treated fruit at CH and CH + 1W).

## 4 Discussion

Environmental factors, mainly light and temperature, among others, have been widely reported to affect anthocyanin content and thus skin blush in apple cultivars (Lancaster, 1992; Ubi et al., 2006; Honda and Moriya, 2018; Musacchi and Serra, 2018; Toivonen et al., 2019; Chen et al., 2021). Particularly concerning light, the use of reflective groundcovers can improve light distribution in the canopy, enhancing the fruit light environment (Privé et al., 2008; Robinson and Gonzalez, 2022; Miah and Farcuh, 2024a). While there are multiple reports indicating that reflective groundcover deployment can increase apple skin blush (Privé et al., 2008; Iglesias and Alegre, 2009; Toivonen et al., 2019; Mupambi et al., 2021; Robinson and Gonzalez, 2022; Kon and Clavet, 2023; Miah and Farcuh, 2024a, 2024b), integrative studies assessing its impact at the environmental, physiological, gene, and metabolite levels are limited. In this work, consistently over two consecutive years, we observed a tendency for an increased red skin coloration (with >60% blush one week before commercial harvest), advanced maturity, upregulated expression of anthocyanin biosynthetic genes, and a correspondingly increased total anthocyanin content, and no effect in fruit drop, for ‘Evercrisp’ fruit where reflective groundcovers had been deployed, under mid-Atlantic environmental conditions. This would allow for an earlier harvest of this late ripening cultivar, which would be packing out in the premium grades as compared to control.

In apples, and in other fruit, light and, particularly, ultraviolet radiation (UV) has been reported to induce anthocyanin biosynthesis and therefore to enhance red skin coloration (Dong et al., 1995; Charles and Arul, 2007; Honda and Moriya, 2018; Toivonen et al., 2019; Miah and Farcuh, 2024b). Consistent with our work, reflective groundcovers deployment has been shown to enhance UV reflectance from the ground into the canopy of the tree with respect to the control (**Tables 1**, **2**), in different locations and cultivars (Toivonen et al., 2019; Kon and Clavet, 2023; Miah and Farcuh, 2024a). Furthermore, the significantly higher PPFD reflectance values for reflective groundcovers observed in this study agree with earlier reports (Privé et al., 2008; Iglesias and Alegre, 2009; Toivonen et al., 2019; Robinson and Gonzalez, 2022; Kon and Clavet, 2023; Miah and Farcuh, 2024a). The improved light environment in the tree canopy that results from reflective groundcover deployment can boost apple biosynthesis of anthocyanins indirectly, through photosynthesis enhancement, which will amplify the quantity of assimilates allocated to sink tissues (i.e., fruit) and therefore supply the substrate needed for biosynthesis of anthocyanins, or directly by inducing the expression and activity of anthocyanin biosynthesis-related genes and enzymes, which have been shown to be increased by light (Zhiqiang et al., 1999; Vimolmangkang et al., 2014; Chen et al., 2021). This particular study is in agreement with the latter, demonstrated by the significant upregulation of all the evaluated anthocyanin biosynthesis-related structural genes (*MdPAL*, *MdCHS*, *MdCHI*, *MdF3H*, *MdDFR*, *MdLDOX*, and *MdUFGT*), the transcription factor *MdMYB10* (**Figure 4**), and the increased total anthocyanin content (**Figure 5**) in the reflective groundcover-treated fruit as compared to the control. It has been shown that there is a marked increase in transcript accumulation of anthocyanin biosynthetic genes in apples treated with high light intensities as compared to low light intensities (An et al., 2020), supporting our results. Work on ‘Ambrosia’ apples subjected to reflective groundcover deployment during on-the-tree ripening displayed comparable results to the current study (Toivonen et al., 2019). However, in locations different than the mid-Atlantic US, as for example in the US Pacific Northwest, an excess of radiation in the PPFD and UV wavelengths can negatively affect apple fruit due to the potential occurrence of sunburn (Mupambi et al., 2021). In the present study, reflective groundcover deployment did not result in the presence of sunburn in ‘Evercrisp’ fruit.

The transcription factor *MdMYB10* is known to be key in regulating the transcript accumulation of anthocyanin biosynthesis-related structural genes and hence on impacting apple red skin coloration (Ban et al., 2007; Espley et al., 2007, 2009; Telias et al., 2011; Ryu et al., 2022). This is consistent with the significantly positive associations obtained in the present study between *MdMYB10* and anthocyanin biosynthesis-related structural genes (**Supplementary Table S2**). Additionally, the positive correlations observed between total anthocyanin content and expression levels of the anthocyanin biosynthesis-related structural genes and *MdMYB10* (**Supplementary Table S2**) are supported by previous studies in apples (Lister et al., 1996; Honda et al., 2002; Toivonen et al., 2019; Miah and Farcuh, 2024b). In fact, the higher transcript accumulation of anthocyanin biosynthetic-related genes corresponded with the increased total anthocyanin content and with the increased red skin coloration in reflective groundcover-treated fruit, supporting the positive and negative correlations of the above parameters with skin blush and surface skin hue angle, respectively (**Supplementary Table S2**). These results are in agreement with previous reports in ‘Ambrosia’, ‘Gala’, and ‘Honeycrisp’ apples submitted to the deployment of reflective groundcovers, and with the apple cultivar ‘Fortune’ exposed to high sunlight (Feng et al., 2013; Overbeck et al., 2013; Toivonen et al., 2019; Miah and Farcuh, 2024b) and can explain the significant increase in red blush in reflective groundcover-treated ‘Evercrisp’ apples [>60% skin blush in the first evaluated ripening stage (1WBCH)], with respect to control fruit [which only reached these values at the last assessed ripening stage (CH + 1W)]. As fruit located at the upper canopy generally reaches a higher and earlier red skin coloration than fruit located at the lower third of the canopy (Layne et al., 2002), the use of reflective groundcovers can help achieve a uniform red skin coloration throughout the tree, while ensuring more fruit that packs out in the premium grades.

In the mid-Atlantic US, and in many other locations, preharvest fruit drop is a key problem affecting several apple cultivars (Irish-Brown et al., 2011; Liu et al., 2022; Miah and Farcuh, 2024a), including ‘Evercrisp’. Earlier reports have indicated that in apple cultivars such as ‘Golden Delicious’ and ‘Honeycrisp’ preharvest fruit drop is significantly enhanced as ethylene production increases (Li et al., 2010; Miah and Farcuh, 2024a). This explains the positive correlation between IEC and preharvest fruit drop observed in the present study (**Supplementary Table S2**). However, reflective groundcover deployment did not increase ‘Evercrisp’ preharvest fruit drop when compared to control (**Figure 1**). This in agreement with what was reported in our previous work in ‘Honeycrisp’ fruit ripening on the tree under mid-Atlantic US environmental conditions (Miah and Farcuh, 2024a). Consequently, the use of reflective groundcovers would ensure that ‘Evercrisp’ fruit in the lower third of the tree canopy can fulfill the minimum acceptable red skin coloration 1 week before commercial harvest without increasing preharvest fruit drop.

Concerning fruit maturity and quality, the effects of the use of reflective groundcovers have been variable among different studies. Although some authors have reported that there are no changes in fruit maturity and quality-related properties of the fruit after reflective groundcover deployment (Iglesias and Alegre, 2009; Privé et al., 2011; Funke and Blanke, 2021; Mupambi et al., 2021; Kon and Clavet, 2023), in the current work, reflective groundcover-treated ‘Evercrisp’ fruit exhibited a more advanced fruit maturity than control fruit, displayed by an increased IEC, decreased flesh firmness and acidity, but enhanced starch degradation and SSC (**Figure 2**), consistent with previous reports in apples (Overbeck et al., 2013; Miah and Farcuh, 2024a) and peaches (Layne et al., 2001). Furthermore, the significantly lower background skin hue angles and I_AD_ values observed in reflective groundcover-treated fruit as compared to control (**Figure 3**) indicate a significant change in background color from green to yellow, which has been associated with increased fruit maturity (Ziosi et al., 2008; Miah et al., 2023). The latter also explains the negative correlation observed between IEC with both background skin hue angles and I_AD_ values (**Supplementary Table S2**), in agreement with earlier findings (Wang and Dilley, 2001; Mahdavi et al., 2022; Miah et al., 2023). We hypothesize that the more advanced fruit maturity in reflective groundcover-treated ‘Evercrisp’ apples in the mid-Atlantic US could be related to an increased air temperature within the lower canopy of the reflective groundcover-treated trees, which could result from the increased light reflectance promoted by this treatment, but these needs further investigation. Furthermore, the inconsistencies among studies regarding reflective groundcover effects on fruit maturity and quality could be explained by the diverse orchard practices, environmental conditions, assessed apple cultivars, and reflective groundcover deployment length employed in the different studies that have been conducted. Moreover, anthocyanin content in apple fruit has been reported to be influenced by ethylene production (Faragher and Brohier, 1984; Blankenship and Unrath, 1988; Wang and Dilley, 2001; Whale and Singh, 2007; Shafiq et al., 2014; Chen et al., 2021; Farcuh et al., 2022; Miah and Farcuh, 2024b). The latter explains the significantly positive associations observed between IEC and the expression of anthocyanin biosynthesis-related structural genes, transcript accumulation of *MdMYB10*, total anthocyanin content, and skin blush, and the significantly negative association observed between IEC and surface skin hue angle in this work (**Supplementary Table S2**). Therefore, our results suggest that reflective groundcover deployment will hasten fruit ripening of ‘Evercrisp’ fruit located in the lower third of the tree canopy while promoting anthocyanin content and thus meeting the minimum red color requirements before commercial harvest. This would allow for an earlier harvest of the reflective groundcover-treated ‘Evercrisp’ fruit as compared to control. Additional work is ongoing to understand any potential impacts of preharvest reflective groundcover deployment on postharvest storage and shelf-life behavior of ‘Evercrisp’ apple fruit.

When looking at the PCA results (**Figure 6**), the positioning of the different combinations of treatments and evaluation periods in component 1 of the PCA can be explained by control ‘Evercrisp’ fruit at 1WBCH presenting the lowest IEC, fruit drop, most delayed fruit maturity, most decreased skin red blush values (<50%), and expression levels for anthocyanin biosynthesis-related genes, and total anthocyanin content. Additionally, reflective groundcover-treated fruit at 1WBCH and control fruit at CH followed, with an intermediary position regarding IEC and fruit maturity, attaining ~60% blush, supported by the higher transcript accumulation of anthocyanin biosynthesis-related genes, and total anthocyanin content, but with control fruit at CH displaying a greater fruit drop. Furthermore, reflective groundcover-treated fruit at CH and control fruit at CH + 1W positioned next, exhibiting a significantly higher IEC, more advanced fruit maturity, displaying >60% blush resulting from an even more enhanced expression of anthocyanin biosynthesis-related genes and total anthocyanin content, yet with control fruit at CH + 1W displaying a higher fruit drop. Lastly, reflective groundcover-treated ‘Evercrisp’ fruit at CH + 1W presented the significantly highest IEC, most hastened fruit maturity, and highest skin red blush values (>80%) together with transcript accumulation of anthocyanin biosynthesis-related genes, and total anthocyanin content. Because these results might only be valid under mid-Atlantic US environmental conditions, future work targeting the impact of reflective groundcovers in multiple apple cultivars grown under diverse regions is continuing.

In conclusion, ‘Evercrisp’ apples subjected to reflective groundcover deployment in the mid-Atlantic US and assessed for 2 years showed significant differences with respect to a control, in terms of light reflectance, IEC, fruit-quality-related physicochemical parameters, skin coloration, expression levels of important anthocyanin biosynthesis-related structural genes and transcription factors, and total anthocyanin content, throughout ripening on the tree. Reflective groundcover-treated fruit displayed an enhanced red skin coloration, reaching >60% blush 1 week before commercial harvest, i.e., 2 weeks earlier than control fruit. This is explained by a significant increased transcript accumulation of anthocyanin biosynthesis-related genes and *MdMYB10*, promoted by an increased PPFD and UV reflection (>5–25 times greater than control) and a higher total anthocyanin content in reflective groundcover-treated apples. Furthermore, ‘Evercrisp’ fruit subjected to reflective groundcover deployment also exhibited an increased IEC and an advanced maturity, but without differences in fruit drop, as compared to control fruit, during on-the-tree ripening. Our work suggests that reflective groundcover deployment in ‘Evercrisp’ fruit grown in the mid-Atlantic US and under the experimental conditions of this study can allow for an earlier harvest (of at least 1 week) of this late ripening cultivar, which will pack out in the premium grades, as compared to a control. This would avoid exposing the fruit to the risk of freezing events, common in the region near ‘Evercrisp’ fruit commercial harvest period, significantly increasing fruit crop value.

## Data Availability

The original contributions presented in the study are included in the article/**Supplementary Material**. Further inquiries can be directed to the corresponding author.
