# Supplementary material for: Reflective groundcovers promote anthocyanin content and advance fruit maturity of ‘Evercrisp’ apples grown in the Mid-Atlantic US
Source: Front Plant Sci. 2024 Oct 24;15:1478498. doi: 10.3389/fpls.2024.1478498 (PMC11540770; doi:10.3389/fpls.2024.1478498)
Supplement: Supplementary file 1 [file Table1.docx]

Supplementary Material

**Supplementary Table S1.** List of primers used for conducting qRT-PCR

| **Gene name** | **Description** | **Primer orientation** | **Primer sequence**  **(5’ to 3’)** |
| --- | --- | --- | --- |
| *MdPAL* | Phenylalanine ammonia-lyase | Forward | GTGCTGTGGAGTCCCCGCTT |
|  |  | Reverse | GGTGA GGCTCTCTCCGCCAAGT |
| *MdCHS* | Chalcone synthase | Forward | GGAGACAACTGGAGAAGGACTGGAA |
|  |  | Reverse | CGACATTGATACTGGTGTCTTCA |
| *MdCHI* | Chalcone isomerase | Forward | GGGATAACCTCGCGGCCAAA |
|  |  | Reverse | GCATCCATGCCGGAAGCTACAA |
| *MdF3H* | Flavanone 3-hydroxylase | Forward | TGGAAGCTTGTGAGGACTGGGGT |
|  |  | Reverse | CTCCTCCGATGGCAAATCAAAGA |
| *MdDFR* | Dihydroflavonol 4-reductase | Forward | GATAGGGTTTGAGTTCAAGTA |
|  |  | Reverse | TCTCCTCAGCAGCCTCAGTTTTCT |
| *MdLDOX* | Leucoanthocyanidin dioxygenase | Forward | CCAAGTGAAGCGGGTTGTGCT |
|  |  | Reverse | CAAAGCAGGCGGACAGGAGTAGC |
| *MdUFGT* | UDP glucose-flavonoid 3- o -glucosyl transferase | Forward | CCACCGCCCTTCCAAACACTCT |
|  |  | Reverse | CACCCTTATGTTACGCGGCATGT |
| *MdMYB10* | Transcription factor | Forward | TGCCTGGACTCGAGAGGAAGACA |
|  |  | Reverse | CCTGTTTCCCAAAAGCCTGTGAA |
| *MdACT* | Actin | Forward | TGACCGAATGAGCAAGGAAATTACT |
|  |  | Reverse | TACTCAGCTTTGGCAATCCACATC |

**Supplementary Table S2.** Pearson correlation coefficients for fruit drop, internal ethylene concentration (IEC), physicochemical parameters, skin coloration, key anthocyanin biosynthesis- related genes, and total anthocyanin concentration in ‘Evercrisp’ apples.

| **Feature** | **Fruit drop** | **IEC** | **Firm-ness** | **SPI** | **SSC** | **TA** | **Surface Hue** | **Skin Blush** | **Back-ground Hue** | **I_AD_** | ***PAL*** | ***CHS*** | ***CHI*** | ***F3H*** | ***DFR*** | ***LDOX*** | ***UFGT*** | ***MYB10*** | **Total Antho-cyanins** |
| --- | --- | --- | --- | --- | --- | --- | --- | --- | --- | --- | --- | --- | --- | --- | --- | --- | --- | --- | --- |
| Fruit drop | 1.00 | 0.91 * | −0.80 * | 0.90 * | 0.82 * | −0.90 * | −0.80 * | 0.72 * | −0.92 * | −0.88 * | 0.89 * | 0.91* | 0.86 * | 0.90 * | 0.90 * | 0.84 * | 0.89* | 0.90 * | 0.61 * |
| IEC |  | 1.00 | −0.93 * | 0.98 * | 0.95 * | −0.95 * | −0.92 * | 0.93 * | −0.97 * | −0.96 * | 0.97 * | 0.98 * | 0.96 * | 0.96 * | 0.97 * | 0.95 * | 0.96 * | 0.98 * | 0.87 * |
| Firmness |  |  | 1.00 | −0.96 * | −0.96 * | 0.95 * | 0.96 * | −0.95 * | 0.93 * | 0.97 * | −0.96 * | −0.94 * | −0.95 * | −0.96 * | −0.94 * | −0.96 * | −0.96 * | −0.95 * | −0.88* |
| SPI |  |  |  | 1.00 | 0.97 * | −0.94 * | −0.95 * | 0.90 * | −0.95 * | −0.93 * | 0.97 * | 0.96 * | 0.96 * | 0.97 * | 0.96 * | 0.95 * | 0.97 * | 0.96 * | 0.84 * |
| SSC |  |  |  |  | 1.00 | −0.93 * | −0.94 * | 0.91 * | −0.92 * | −0.95 * | 0.95 * | 0.93 * | 0.92 * | 0.95 * | 0.93 * | 0.92 * | 0.94 * | 0.94 * | 0.81 * |
| TA |  |  |  |  |  | 1.00 | 0.96 * | −0.90 * | 0.95 * | 0.97 * | −0.95 * | −0.94 * | −0.93 * | −0.95 * | −0.95 * | −0.92 * | −0.95 * | −0.95 * | −0.81 * |
| Surface Hue |  |  |  |  |  |  | 1.00 | −0.96 * | 0.95 * | 0.98 * | −0.94 * | −0.93 * | −0.95 * | −0.94 * | −0.93 * | −0.96 * | −0.94 * | −0.94 * | −0.89 * |
| Skin Blush |  |  |  |  |  |  |  | 1.00 | −0.91 * | −0.94 * | 0.90 * | 0.90 * | 0.93 * | 0.89 * | 0.90 * | 0.95 * | 0.90 * | 0.90 * | 0.93 * |
| Background Hue |  |  |  |  |  |  |  |  | 1.00 | 0.98 * | −0.96 * | −0.97 * | −0.96 * | −0.96 * | −0.97 * | −0.94 * | −0.96 * | −0.97 * | −0.82 * |
| I_AD_ |  |  |  |  |  |  |  |  |  | 1.00 | −0.97 * | −0.96 * | −0.95 * | −0.97 * | −0.96 * | −0.96 * | −0.96 * | −0.96 * | −0.83 * |
| *PAL* |  |  |  |  |  |  |  |  |  |  | 1.00 | 0.96 * | 0.95 * | 0.97 * | 0.96 * | 0.94 * | 0.97 * | 0.97 * | 0.82 * |
| *CHS* |  |  |  |  |  |  |  |  |  |  |  | 1.00 | 0.96 * | 0.96 * | 0.97 * | 0.95 * | 0.96 * | 0.97 * | 0.83 * |
| *CHI* |  |  |  |  |  |  |  |  |  |  |  |  | 1.00 | 0.95 * | 0.96 * | 0.96 * | 0.95 * | 0.96 * | 0.89 * |
| *F3H* |  |  |  |  |  |  |  |  |  |  |  |  |  | 1.00 | 0.96 * | 0.94 * | 0.96 * | 0.97 * | 0.81 * |
| *DFR* |  |  |  |  |  |  |  |  |  |  |  |  |  |  | 1.00 | 0.95 * | 0.96 * | 0.97 * | 0.84 * |
| *LDOX* |  |  |  |  |  |  |  |  |  |  |  |  |  |  |  | 1.00 | 0.95 * | 0.95 * | 0.89 * |
| *UFGT* |  |  |  |  |  |  |  |  |  |  |  |  |  |  |  |  | 1.00 | 0.97 * | 0.94 * |
| *MYB10* |  |  |  |  |  |  |  |  |  |  |  |  |  |  |  |  |  | 1.00 | 0.85 * |
| Total Anthocyanins |  |  |  |  |  |  |  |  |  |  |  |  |  |  |  |  |  |  | 1.00 |

All correlations shown are significant (*; P ≤0.05).
